# 3D vascularised proximal tubules-on-a-multiplexed chip model for enhanced cell phenotypes†

**DOI:** 10.1039/d2lc00723a

**Published:** 2023-06-21

**Authors:** Miguel Carracedo, Sanlin Robinson, Babak Alaei, Maryam Clausen, Ryan Hicks, Graham Belfield, Magnus Althage, Annette Bak, Jennifer A. Lewis, Pernille B. L. Hansen, Julie M. Williams

**Affiliations:** a Bioscience Renal, Research and Early Development, Cardiovascular, Renal and Metabolism (CVRM), BioPharmaceuticals R&D, AstraZeneca Gothenburg Sweden juliem.williams@astrazeneca.com; b Wyss Institute for Biologically Inspired Engineering, Harvard University Boston MA USA; c Translational Genomics, Discovery Biology, Discovery Sciences, R&D, AstraZeneca Gothenburg Sweden; d BioPharmaceuticals R&D Cell Therapy, Research and Early Development, Cardiovascular, Renal and Metabolism (CVRM), AstraZeneca Gothenburg Sweden; e School of Cardiovascular and Metabolic Medicine and Sciences, King's College London London UK; f Research and Early Development, Cardiovascular, Renal and Metabolism, BioPharmaceuticals R&D, AstraZeneca Gothenburg Sweden; g Pharmaceutical Sciences, R&D, AstraZeneca Waltham USA

## Abstract

Modelling proximal tubule physiology and pharmacology is essential to understand tubular biology and guide drug discovery. To date, multiple models have been developed; however, their relevance to human disease has yet to be evaluated. Here, we report a 3D vascularized proximal tubule-on-a-multiplexed chip (3DvasPT-MC) device composed of co-localized cylindrical conduits lined with confluent epithelium and endothelium, embedded within a permeable matrix, and independently addressed by a closed-loop perfusion system. Each multiplexed chip contains six 3DvasPT models. We performed RNA-seq and compared the transcriptomic profile of proximal tubule epithelial cells (PTECs) and human glomerular endothelial cells (HGECs) seeded in our 3D vasPT-MCs and on 2D transwell controls with and without a gelatin–fibrin coating. Our results reveal that the transcriptional profile of PTECs is highly dependent on both the matrix and flow, while HGECs exhibit greater phenotypic plasticity and are affected by the matrix, PTECs, and flow. PTECs grown on non-coated Transwells display an enrichment of inflammatory markers, including TNF-a, IL-6, and CXCL6, resembling damaged tubules. However, this inflammatory response is not observed for 3D proximal tubules, which exhibit expression of kidney signature genes, including drug and solute transporters, akin to native tubular tissue. Likewise, the transcriptome of HGEC vessels resembled that of sc-RNAseq from glomerular endothelium when seeded on this matrix and subjected to flow. Our 3D vascularized tubule on chip model has utility for both renal physiology and pharmacology.

## Abstract 2

Incremental increases in complexity in a model of the vascularised proximal tubule give rise to increased maturity and improved physiology.

## Graphical abstract

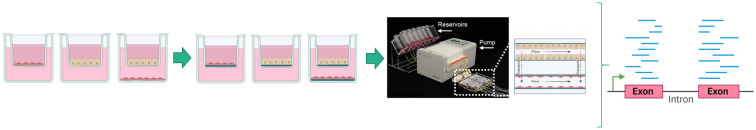

## Introduction

The proximal tubules of the kidney are essential for maintaining homeostasis and reabsorbing water and solutes as well as actively secreting metabolites. These functions are carried out by highly specialized epithelial cells, known as proximal tubule epithelial cells (PTECs) that are present in tubules surrounded by a network of blood vessels lined with endothelial cells (peritubular capillaries). Damage or phenotypic changes to either of these cell types leads to alterations in secretion of drugs and organic ions as well as the reabsorption of water and solutes that can exacerbate kidney disease.^1^ To both avoid drugs that induce injury and design drugs that restore these crucial functions, a faithful replication of the proximal tubule structure and physiology is needed. Whilst *in vivo* animal models are useful, the lack of translation from human to other species substantially hinders both toxicity testing and drug development.

To date, multiple models have been developed to mimic the physiology of proximal tubules. The use of primary cultures and immortalized PTECs have proven useful in attaining basic knowledge of cell biology;^2^ however, due to cellular de-differentiation and loss of key transporters, they do not fully recapitulate PTEC physiology. To capture the complex metabolic, structural, and absorptive functions of the proximal tubule, both microfluidic and 3D bioprinted models have recently been introduced,^3^ including 3D vascularized tubule models capable of both albumin uptake and glucose reabsorption.^4,5^ Perfusable 3D models that enable the co-culture of PTECs and human glomerular microvascular endothelial cells (HGECs) seeded within adjacent open lumens embedded within an extracellular matrix (ECM) composed of gelatin–fibrin are of particular interest.^6^ Despite these recent advances, a comparative study between monocultures, co-cultures, and 3D vascularized proximal tubule models is lacking. Here, we elucidate the role of a 2D *vs.* 3D microenvironment, extracellular matrix, and perfusive flow on transcriptional differences between PTECs and endothelial cells in mono- and co-culture conditions. Our RNA-seq data analysis reveals that the PTEC transcriptional profile is highly dependent on presence of both the gelatin–fibrin matrix and flow, while endothelial cells exhibit a more plastic phenotype that is affected by matrix, flow, and co-culture with PTECs.

## Results

### Cell culture effects on PTEC and HGECs transcriptional profile

**Fig. 1:**
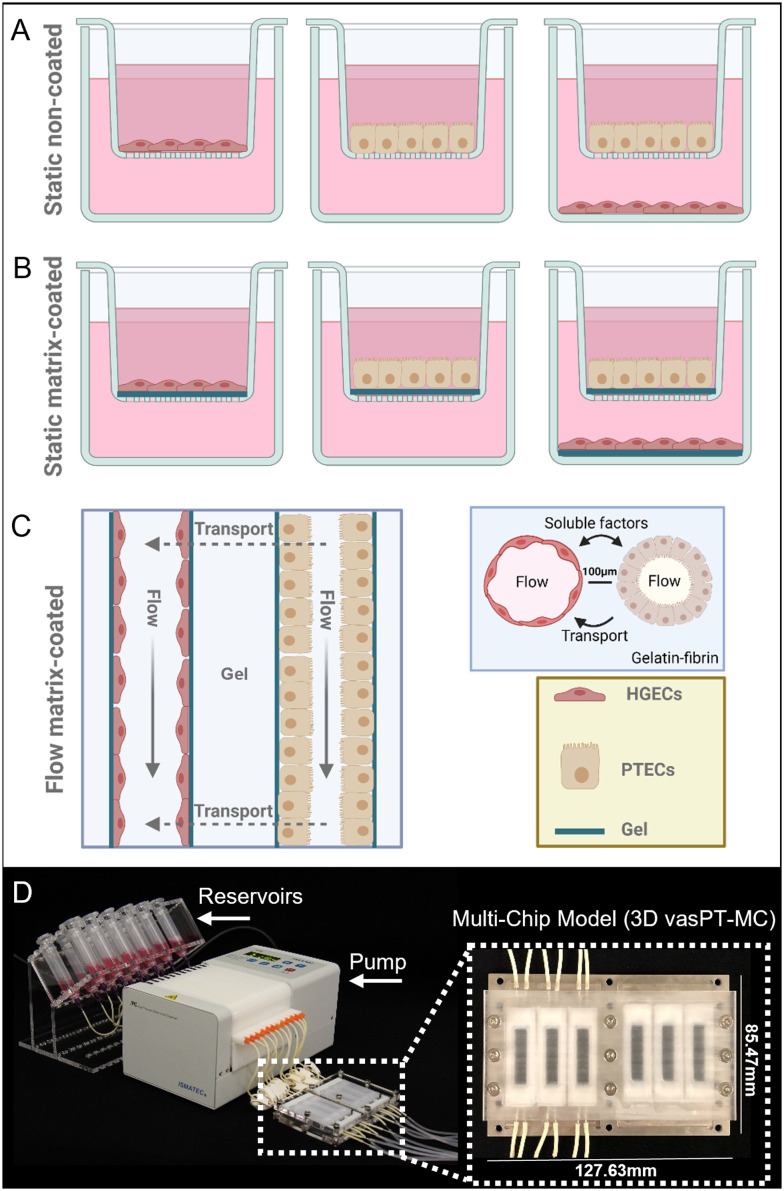
Proximal tubule models in two- and three-dimensions. A) Schematic view of proximal tubule epithelial cells (PTECs) cultured on a transwell membrane (static, non-coated) in the absence and presence of human glomerular endothelial cells (HGECs). B) Schematic view of proximal tubule epithelial cells cultured on a transwell membrane coated with an estimated 1 mm gelatin–fibrin coating matrix (static, coated) in the absence and presence of endothelial cells. C) Schematic views of co-localized 3D blood vessel and proximal tubule embedded within a gelatin–fibrin matrix within 100 μm in a perfusable multichip model. D) Images of the perfusable multi-chip model platform, which shows the media reservoirs, peristaltic pump, and 3D vasPT-MC system. Each device contains 6 individually perfusable 3DvasPT chips, as shown in the magnified image. In all cases, HGECs are seeded and grown on a thin layer of laminin 521 in the absence or presence of matrix. Figure was created utilizing biorender.

To study the impact of different culture conditions on the transcriptional profiles of PTECs and HGECs, cells are seeded on a non-coated or gelatin–fibrin matrix-coated Transwells® (controls) or within tubular and vascular channels embedded within this matrix on our 3D vascularized proximal tubule-on-a-multiplexed chip (3DvasPT-MC) model (Fig. 1). In all cases, HGECs are seeded and grown on a thin layer of laminin 521 in the absence or presence of this underlying matrix. For the coated Transwell model, the gelatin–fibrin matrix is roughly 1 mm thick. The 3DvasPT-MC device is designed and fabricated based on our original single-chip method^6^ (Fig. 1C and D and S1†). Each device contains six 3DvasPT chips with the same outer dimensions as a six-well plate (127.63 mm × 85.47 mm) to facilitate simultaneous imaging during their longitudinal culture. This autoclavable device consists of a supportive base, 3D printed silicone chambers that house the six 3DvasPT models, and top lid that fully encloses the devices to create a 3D tissue microenvironment (Fig. S1†).

**Fig. 2:**
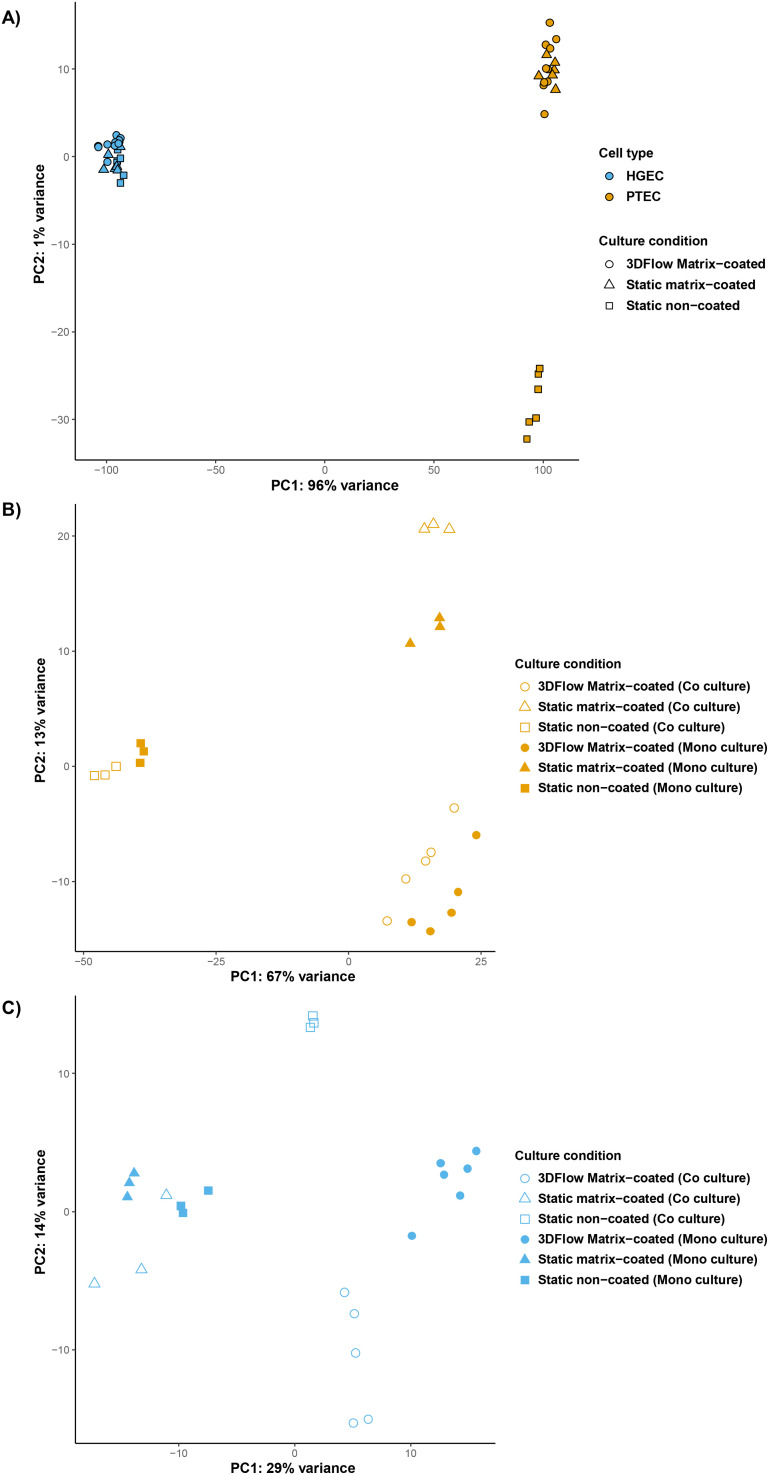
PTEC and HGECs transcriptional profiles depend on cell culture conditions. (A) Principal component analysis (PCA) plot of PTECs and HGECs in different 2D and 3D culture conditions. (B) PCA plot for PTECs in different mono- and co-culture conditions based on their transcriptional profiles. (C) PCA plot for HGECs in different mono- and co-culture conditions based on their transcriptional profiles.

Principal component analysis (PCA) of the transcriptional profiles revealed self-organization among cell types. Interestingly, PTECs divided into two clusters. One cluster formed by PTECs grown on the gelatin–fibrin matrix in either Transwells or on 3D chips and another grown on non-coated Transwells (Fig. 2A). PCA plots of both cell types showed a separation of PTECs into three distinct groups based on the conditions in which cells were grown. The presence of extracellular matrix induced the largest transcriptional changes followed by flow, which had a moderate effect on the transcriptional profile, as compared with static, non-coated conditions. Interestingly, cell co-culture had a lesser impact on gene expression in PTECs (Fig. 2B). By contrast, HGECs exhibited a more homogenous clustering (Fig. 2A) with those grown under static conditions on either matrix-coated or non-coated substrates displaying similar transcriptional signatures. However, the profiles of HGECs subjected to flow on 3D chips were affected more strongly than by co-culturing these cells alongside PTECs (Fig. 2C).

### PTECs more closely resemble *in vivo* tubular transcriptional profile with increasing culture complexity

**Fig. 3:**
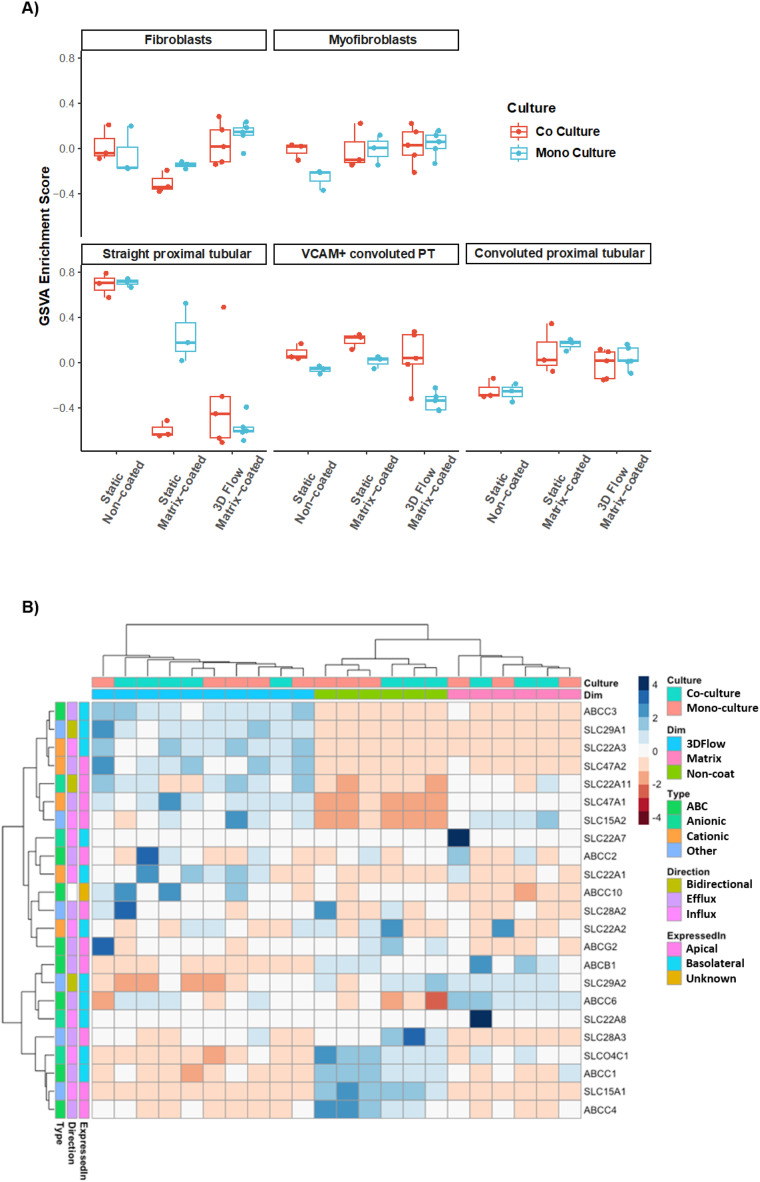
Kidney cell type and transporter enrichment of PTECs grown under different culture conditions. A) Gene set variation analysis between the gene sets from the different PTEC culture conditions and the gene sets from Young *et al.* single-cell RNA-seq. PT: Proximal tubular. B) Heatmap representing absolute gene expression values of selected transporters in PTECs under different culture conditions.

To further understand the phenotypic differences of PTECs cultured under different conditions, we performed gene set variation analysis (GSVA) against a publicly available single cell RNA dataset of the kidney.^7^ While dramatic changes are not observed, GSVA revealed that PTECs grown under static, non-coated, monoculture conditions in Transwells exhibited a gene expression profile more like proximal tubule segment 3 (the straight region between the convoluted proximal tubule and the Loop of Henlé) (Fig. 3A).

Phenotypic differences observed in the GSVA are more evident when exploring the expression profile of different transporters. Mapping renal transporters into the dataset showed that channels are enriched relative to increasing complexity of the culture system, specifically under flow conditions (Fig. 3B).

### PTEC grown on static, non-coated substrates express genes associated with disease and inflammation

**Fig. 4:**
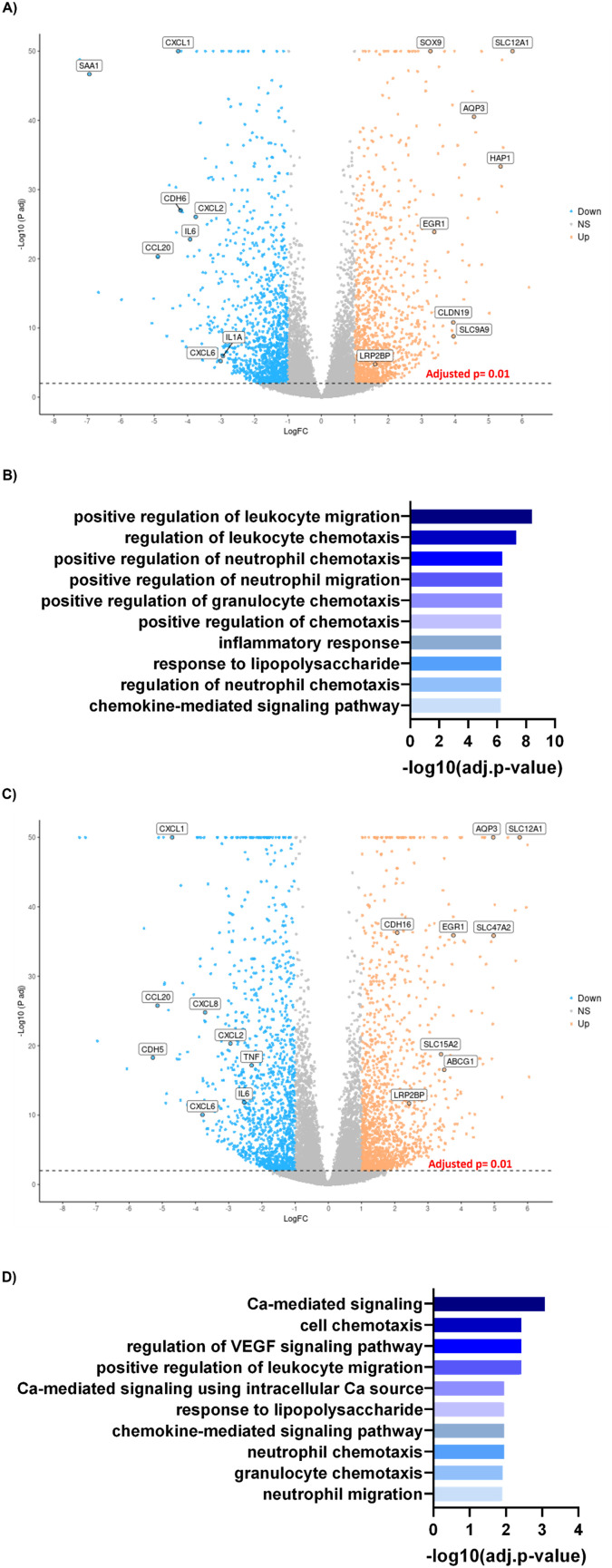
PTECs on chip exhibit less inflammatory genes. A) Volcano plot representing the magnitude of change (logged fold change (logFC)) *versus* the statistical significance (−log_10_(*P*-value)) of all measured genes between PTECs grown coated and non-coated Transwells. Each dot represents a gene meeting the significance threshold. Blue represents down regulation and orange upregulation with respect to static coated and non-coated conditions. B) Gene ontology pathways of the top 100 downregulated genes under static matrix-coated compared with static non-coated conditions ranked by significance. C) Volcano plot of all measured genes between PTECs on chip (flow) *vs.* non-coated Transwell (static) conditions. Blue represents down regulation, while orange denotes upregulation with respect to on chip *versus* static conditions. D) Gene ontology pathways of the top 100 downregulated genes between PTECs on chip *versus* those cultured on static, non-coated Transwells ranked by significance.

Based on the phenotypic differences observed, we next explored the most differentially expressed genes in PTECs grown in monoculture under different substrate conditions (Fig. 4A and C). PTECS grown under static, matrix-coated substrates exhibited a substantial number of differentially downregulated genes compared to those grown under static, non-coated conditions (Fig. 4A). Indeed, the top one hundred significantly downregulated genes reveal changes in pathways related to inflammation and leukocyte migration (Fig. 4B). Particularly, these pathways are associated with genes belonging to the CXC chemokine family, such as CXCL1, CXCL2 and CXCL6, as well as inflammatory molecules like TNF-α and IL-6. The top one hundred significantly downregulated genes in PTECs cultured on 3D chips under flow, matrix-coated conditions exhibit an enrichment in pathways related to calcium-mediated signalling as well as leukocyte chemotaxis, when compared with matrix non-coated controls (Fig. 4D).

**Fig. 5:**
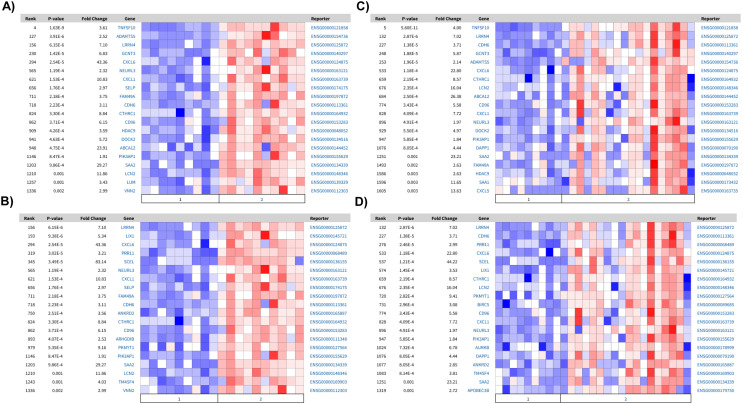
Comparison of the top 100 differentially expressed genes with RNAseq of human tubulointerstitial biopsies. Heatmaps of differentially expressed genes between healthy and FSGS or DN, which present a significant overlap among the top 100 down regulated genes in: matrix-coated *vs.* non-coated in A) healthy (1) *vs.* DN (2): *P*-value: 1.61 × 10^−8^, *Q*-value: 4.84 × 10^−5^, odds ratio: 5.6, size: 20 genes; and B) healthy (1) *vs.* FSGS (2): *P*-value: 1.04 × 10^−5^, *Q*-value: 0.006, odds ratio: 4.2, size: 16 genes. In 3D flow matrix-coated *vs.* non-coated C) healthy (1) *vs.* DN (2): *P*-value: 9.59 × 10^−9^*Q*-value: 7.51 × 10^−5^ odds ratio: 5.8 size: 20 genes and D) healthy (1) *vs.* FSGS (2): *P*-value: 9.59 × 10^−9^*Q*-value: 9.39 × 10^−5^ odds ratio: 5.8 size: 20 genes.

To understand whether these upregulated pathways are biologically relevant, we next mapped the top one hundred upregulated genes in PTECs cultured on static, non-coated substrates against the differentially expressed genes between diseased and healthy tubulointerstitial biopsies from publicly available sources (https://www.nephroseq.org). The upregulated genes under these conditions are associated with the expression profile of diseased tubulointerstitial biopsies, specifically from diabetic nephropathy (DN) and focal segmental glomerulosclerosis (FSGS) patients (Fig. 5A–D). Among these upregulated genes, nine (LRRN4, CDH6, LCN2, CXCL6, CD96, CXCL1, NEURL3, PIK3AP1, SAA2) are present in all datasets. This association with disease is absent when PTECs are cultured on the extracellular matrix under both static (2D) and flow (3D) conditions. Importantly, the genes upregulated for 3D PTECS on chip compared to static, matrix-coated substrates are associated with the downregulated genes in the tubulointerstitial biopsies from FSGS patients compared with healthy living donors (*P*-value: 0.001, *Q*-value: 0.095, odds ratio: 2.9, size: 14 genes).

### Transcriptional profile of HGECs cultured on 3D chips resemble peritubular endothelial cells

**Fig. 6:**
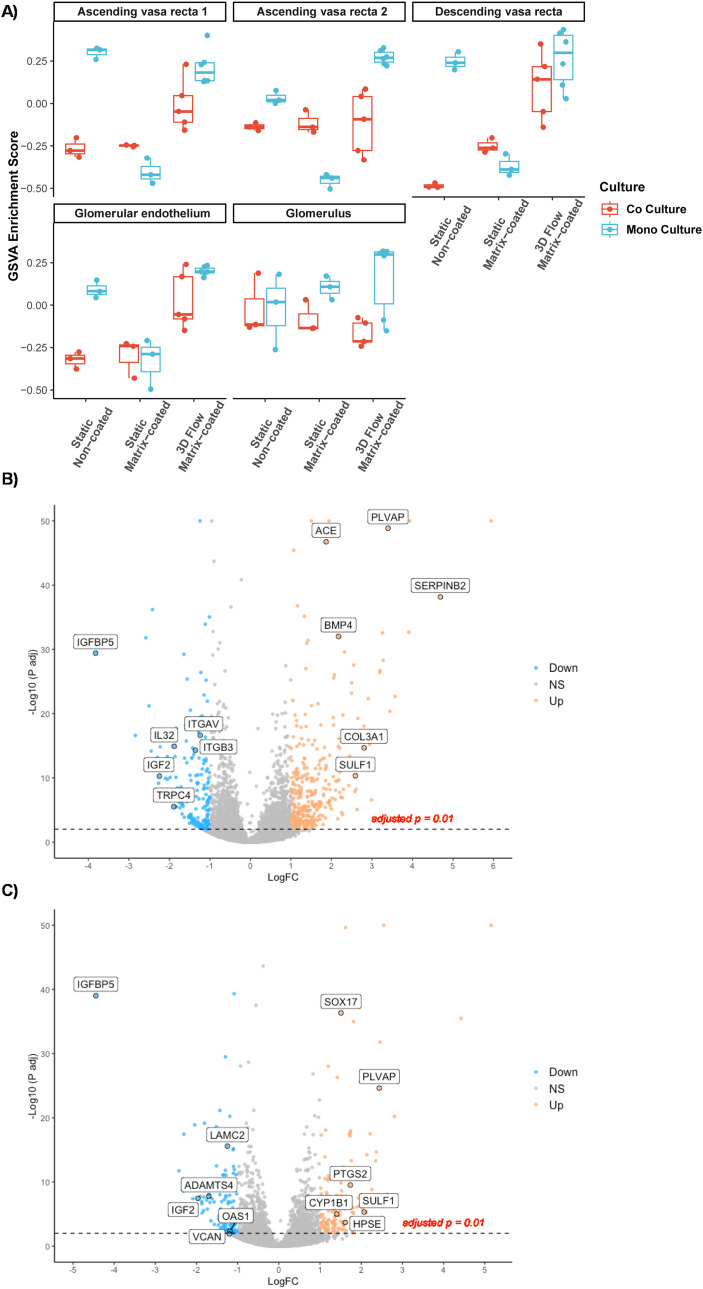
Kidney cell type enrichment profile of HGECs grown under different conditions. A) Gene set variation analysis between the gene sets from the different HGEC culture conditions and the gene sets from Young *et al.* single-cell RNA-seq. AV1 and AV2 for ascending vasa recta, DV for descending vasa recta, G for Glomerulus, GE for glomerular endothelium. B and C) Volcano plots representing the logged fold change (logFC) *vs.* the −log_10_(*P*-value) of all measured genes. Each dot represents a gene meeting the significance threshold. Blue represents down regulation and orange upregulation with respect to PTECs on chip compared to matrix-coated (B) and non-coated (C) Transwells, respectively.

Renal endothelial cells from different areas of the kidney are highly heterogenous in their function and transcriptional profile. To elucidate the role of different culture conditions and co-culture on the phenotype of HGECs, we mapped the differential expression profiles to the gene expression profiles obtained from human kidney single cell RNA-seq. As illustrated in the PCA plot (Fig. 2C) any alteration of the culture conditions led to changes in HGECs expression profile with no discernible phenotype (Fig. 6A). This may be reflective of a generalised increase in endothelial features of the cells when exposed to a richer environment. Interestingly, under flow, HGECs exhibit an enrichment the peritubular capillary marker PLVAP, when compared to both static matrix-coated (Fig. 6B) and non-coated (Fig. 6C) conditions, suggesting a closer resemblance with peritubular endothelial cells.

**Fig. 7:**
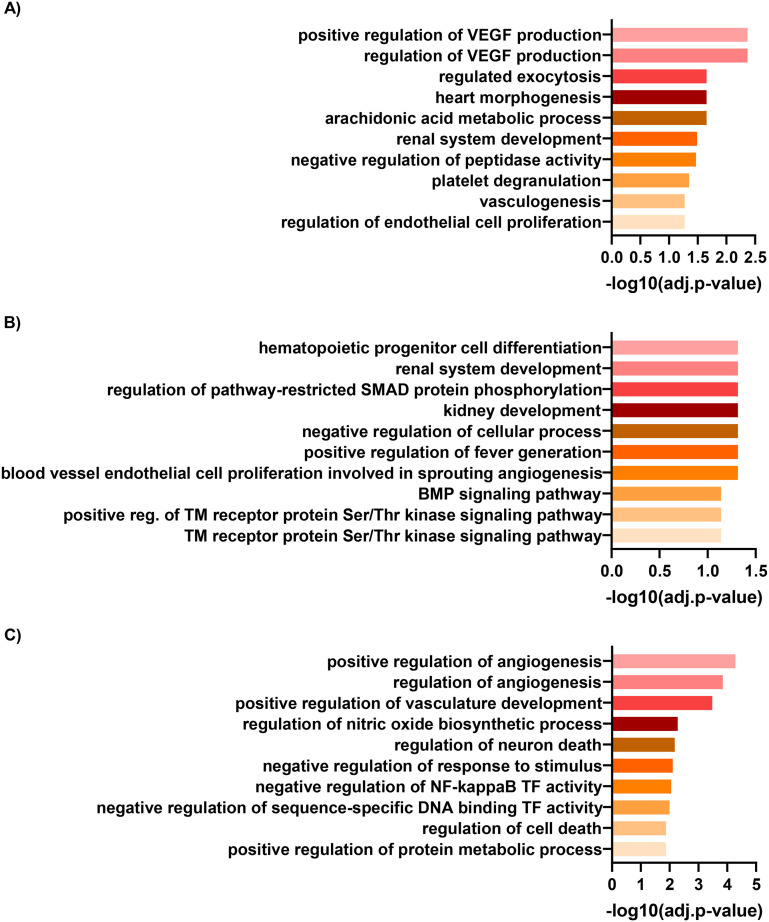
HGEC gene ontology. Gene ontology analyses of the top one-hundred upregulated genes in HGECs between cells cultured on chip under flow compared to A) non-coated Transwells, B) matrix-coated Transwells and C) non-coated Transwells in co-culture conditions.

To understand the pathways underlying the observed enrichment of endothelial genes, we analysed the GO terms of the top one hundred upregulated genes to facilitate direct comparison. HGECs cultured on 3D chips under flow (matrix-coated) showed an enrichment in GO terms related with vascular endothelial growth factor and renal development when compared with either static, non-coated or static matrix-coated conditions, respectively (Fig. 7A and B). In addition, HGECs in co-culture on 3D chips under flow (matrix-coated) compared with static, non-coated conditions, revealed GO terms associated with angiogenesis and vascular development (Fig. 7C).

Finally, we used the comparison analysis tool in the ingenuity pathway analysis software (IPA) applied across the top one hundred up and down regulated genes between cells cultured on 3D chips under flow (matrix-coated) compared to those on static, matrix-coated, non-coated, and non-coated in co-culture conditions, which revealed the same predicted upstream regulators: ERBB3, CEBPB, IFNA, IFNG and BMP2. Among these, ERBB3 was predicted to be the most inhibited, whereas CEBPB the most activated upstream regulator (Table 1). Several genes affected by CEBPB were significantly upregulated in all three comparisons: CYP1A1, HAS2 and FABP4. Of which, CYP1A1 was the single most upregulated gene in monoculture and the second most in coculture. Suggesting CYP1A1's role in flow-mediated endothelial cell fate.

**Table 1:** Predicted upstream regulators

Upstream regulators	3D *vs.* plastic monoculture	3D *vs.* gel monoculture	3D *vs.* plastic co culture
ERBB3	−2961	−2611	−2
CEBPB	1564	2929	2202
IFNA	−2061	−2582	−1951
IFNG	−1937	−2872	−1603
BMP2	2374	2029	1937

## Discussion

This is the first study to present a comprehensive transcriptional comparison of different proximal tubule cultures. Specifically, comparing the transcriptional profile of PTECs and HGECs grown alone or in co-culture under different culture conditions. There are four key findings.

1) PTEC transcriptional profile is highly dependent on culture substrate, whereas HGECs presented a more plastic transcriptional profile, which is dependent on both flow and co-culture. Our results in PTECs are consistent with previous reports in the human proximal tubular epithelial cell line, HK-2, which revealed only 99 differentially expressed genes between monoculture and co-culture with the human microvascular endothelial cell line HMEC-1.^8^ Nonetheless, despite the similarities in transcriptional profiles between monoculture and co-culture, co-culture of PTECs with microvascular endothelial cells led to an increase in ZO-1 expression, cell density, and mitochondrial activity.^4^ This suggests that endothelial cells might alter PTEC phenotype through transcriptionally independent mechanisms. On the other hand, the phenotypic plasticity of HGECs observed in our models is consistent with the heterogeneity of endothelial cells within tissues,^9^ specifically in the kidney where 24 endothelial cell phenotypes have been described in mice^10^ and three distinct types in human biopsies.^11^

2) GSVA revealed that PTECs acquire a more mature phenotype, resembling the transcriptional profile of tubule with increased culture complexity. This observation is in line with the increased microvilli complexity as well as ECM remodelling, a characteristic of mature PTECs, previously reported in this 3D vascularized model.^6^ Moreover, our finding, although previously inferred in preceding studies, is of particular importance considering that the different tubular segments present different transcriptional profiles^12^ and therefore, contrasting functions. One essential feature determining the functionality of proximal tubule cells is the expression of channels and transporters capable of secreting drugs and organic ions and reabsorbing water and solutes. Importantly, previous studies, utilizing microfluidic and bioprinted devices, have successfully shown SLC22A2 (ref. 13) and SLC5A2 (SGLT2)^4,6^ functionality. Here, we build on these observations by providing a comprehensive expression pattern of the different transporters across different culture conditions. In fact, our unsupervised hierarchical clustering of transporters revealed that culturing PTECs on matrix-coated conditions under flow enhanced gene expression. It remains to be determined if these are functional under these conditions. Co-culture with HGECs did not alter the expression pattern of these transporters; reinforcing previous observations where co-culture of HGECs with PTECs did not alter glucose reabsorption.^6^

3) In parallel, our analysis of the top differentially upregulated genes revealed that culture of PTECs in non-coated conditions promotes inflammatory transcriptional profiles, characteristic of FSGS and DN. Specifically, we discovered nine genes consistently upregulated in the PTECs grown under non-coated conditions as well as in both the FSGS and DN tubulointerstitial biopsies. Among these genes, and in accordance with the gene ontology analyses, several of these were chemokines. Moreover, lipocalin 2 (LCN2), is commonly used as a biomarker of drug-induced kidney injury^14^ and is associated with renal disease progression.^15^ Overall, this data further reinforces the relationship between inflammation and the gene expression profile of PTECs grown in static non-coated conditions. Importantly, no association with DN or FSGS is found when PTECs are grown under matrix-coated conditions. Therefore, the association between gene expression signatures of PTECs grown under flow matrix-coated conditions and healthy biopsies suggests that culture of PTECs on a matrix prevents the expression of disease associated markers, whereas flow further enhances the expression of genes associated with healthy renal tissue. Overall, these matrix-mediated observed effects suggest that gelatin–fibrin provides the appropriate stiffness (∼4 kPa, akin to the kidney cortex) necessary for PTECs to express a healthier phenotype. This phenotypic plasticity is further reinforced by the fact that, despite being grown in plastic for initial expansion, PTECs quickly re-adapted to their surrounding environment, as shown by the transcriptional changes, once more physiological conditions are applied.

4) Finally, GSVA of the transcriptional profile of HGECS demonstrated that HGECs grown under flow matrix-coated conditions, when compared with all static, expressed the peritubular capillary marker PLVAP. Revealing that, despite HGECs being isolated from decapsulated glomeruli and the transcriptional heterogeneity observed in all the conditions, growing HGECs under flow matrix-coated conditions promotes a phenotypic plasticity in microvascular endothelial cells, resembling more faithfully the transcriptional profile of the human kidney. Moreover, closer analysis of the data revealed CEBPB as a predicted upstream regulator. This result is in accordance with a previous report where, utilizing a systems biology approach, CEBPB is found to be downregulated in oscillatory shear *vs.* pulsatile shear. More importantly, CEBPB has been postulated to be a regulator of endothelial cell homeostasis in the early hours of shear response.^16^ In our data, among all the predicted downstream genes of CEBPB: CYP1A1, FABP4 and HAS2 are upregulated in all three comparisons. In fact, CYP1A1 is the among the most upregulated gene in all three culture conditions. Interestingly, CYP1A1 has been implicated in polyunsaturated fatty acid metabolism,^17^ and CYP1A1 polymorphisms have been associated with chronic kidney disease of unknown aetiology.^18^ Likewise, the other members of the CEBPB pathway have a relevance in endothelial function. FABP4 is a regulator of endothelial cell proliferation^19^ and HAS2 is a key enzyme in the synthesis of the glycocalyx. The expression of HAS2 is known to be upregulated by laminar shear^20^ leading to angiogenic sprouting.^21^ Taken together, these pathways reinforce the observed GO terms, suggesting that HGECs under flow in matrix coated conditions promotes HGECs maturation.

## Conclusions

In summary, we demonstrate how variable and plastic the transcriptional profiles of PTECs and HGECs are under different culture conditions. We show that healthy epithelial and endothelial cell phenotypes are obtained in our 3DvasPT model due to its more physiologically relevant microenvironment that encompasses co-localized tubules and vessels embedded in matrix and perfused *via* flow on chip. We also demonstrate an initial step towards multiplexed models that address the need for higher throughput for drug testing. By contrast, we find that care must be taken when interpreting findings using simple 2D culture models, since even human primary cells exhibit a strong disease phenotype under such conditions. Returning cells to basal levels after an injury does not reflect a restoration of health, but merely a reconstitution of the abnormal phenotype displayed under these non-physiological conditions. Our findings shed new light on the lack of translatability of *in vitro* 2D models to clinical studies and further underscore the need for 3D physiologically relevant models for both drug development and testing.

## Materials and methods

### Cell culture

Human immortalized PTECs (RPTEC/TERT1; ATCC CRL4031) were cultured using the supplier's specified protocol with some modifications. The cell media was modified to contain DMEM F-12 without glucose (pH 7.3 ± 0.05) (ATCC, 30-2006), NaHCO_3_ (1.2 mg mL^−1^) (Sigma-Aldrich, S5761), d-glucose (100 mg dL^−1^) (Sigma-Aldrich, G7021), ITS (1× concentration, 13146-5ML; Sigma), triiodothyronine (5 pM) (Sigma-Aldrich, T6397), sodium selenite (3.65 ng mL^−1^) (Sigma-Aldrich, S5261), PGE1 (25 ng mL^−1^) (Cayman, 13010), hydrocortisone (25 ng mL^−1^) (Sigma-Aldrich, H0888), ascorbic acid (3.5 μg mL^−1^) (Sigma-Aldrich, A92902), and EGF (10 ng mL^−1^) (R&D systems, 236-EG). The PTECs were cultured up to passage 20.

Glomerular microvascular endothelial cells (HGECs, human primary; Cell Systems) from decapsulated glomeruli isolated from normal human kidney cortical tissue, were cultured utilizing complete classic medium with 10% serum and CultureBoost (Cell systems, 4Z0-500) according to the supplier's specified protocol. The HGECs were cultured up to passage 8 and then plated onto laminin 521 coating (10 μg ml^−1^).

### 3D vascularized PT-on-multiplexed chip model

A multiplexed 3D vascularized proximal tubule model was designed and fabricated based on our original single-chip protocol^6^ (Fig. 1 and S1†). We chose to modify our previous single-chip device to increase our experimental throughput. Specifically, each 3D vascularized proximal tubules-on-a-multiplexed chip (3DvasPT-MC) consisted of six individually perfusable tubules. To easily adapt the 3DvasPT-MC to pre-existing plate holders, such as those found on a standard BF microscope, we designed the 3DvasPT-MC to have the same outer dimensions as a six-well plate (127.63 mm × 85.47 mm). This choice greatly reduced the daily imaging time as all six chips could be imaged and put back into the incubator together, instead of the previous design which required us to remove and return each chip individually. The autoclavable 3DvasPT-MC platform was composed of a supportive base, 3D printed silicone chambers that house each 3DvasPT model (6 per 3DvasPT-MC), and a lid to enclose the tissue microenvironment (Fig. S1†). The supportive base was machined from a 3 mm stainless-steel plate by milling two insets for holding and aligning glass slides (75 mm × 50 mm × 1 mm) and six cut-outs for visualizing the individual tissue chambers. The rectangular silicone chambers were printed using a custom-built 3D printer (ABG 10000, Aerotech Inc., Pittsburgh, PA, USA) directly onto the glass slides using a silicone ink composed of a two-part elastomer (SE 1700 Dow Chemical) with a 10 : 1 base to catalyst ratio (by weight). Prior to printing, this ink homogenized using a mixer (AE-310, Thinky Corp., Japan), loaded into an ink reservoir (EFD Inc., East Providence, RI, USA), and centrifuged to remove any air bubbles. Upon printing, the silicone chambers were cured in the oven at 80 °C for 4 h. Subsequently, parts were washed in a mild detergent/water mixture for several hours, rinsed, air dried, then autoclaved. The lid to enclose the tissue microenvironment was machined from polycarbonate, its length was the same as the steel plate below so that it could be screwed into the plate sandwiching the silicone chambers. The surface of polycarbonate lid was milled flat for even contact with the silicone chambers enabling a good seal.

We fabricated two channels in each of the six silicone tissue chambers by inserting metal pins into opposing side walls of the silicone chamber and then placing wire (diameter ∼150 μm) down one pin and out through the opposite pin (Fig. S1†) (6). Next, we pipetted a gelatin–fibrinogen solution into each of the chambers and cured them for 45 min in the incubator at 37 °C (6). We carefully removed the wires leaving behind open channels. The individually addressable vascular and PT channels were connected to an external perfusion device that houses the media reservoirs (Fig. 1 and S1†). The two individually addressable channels were perfused with PTEC and endothelial media, respectively, for a minimum of 4 h. Each channel was then seeded with PTECs or HGECs at respective concentrations of approximately 2 × 10^7^ cells per mL. When PTECs and HGECs were co-cultured on chip, the epithelial cells were seeded first and allowed to grow to confluency prior to introducing the endothelial cells to the adjacent channel (Fig. S2†). The endothelial channel was first coated with recombinant human laminin 521 (lam521; Biolamina) by injecting a laminin solution (10 μg mL^−1^) into the open lumen (no cells) and leaving it undisturbed for 2 h under ambient conditions or 1 h at 37 °C, if PTECs were already seeded in the adjacent channel. HGECs were then seeded within the laminin-coated channel and the 3D chip was placed in an incubator under static conditions for at least 4 h to ensure that the cells adhered to the channel walls (*i.e.*, the cylindrical interface between the open lumens and the surrounding gelatin–fibrin matrix). The appropriate media was then perfused through each channel at a rate of 200 μL min^−1^ using a peristaltic pump prior to being returned to its original reservoir(s). The media was changed every 48 h.

As controls, PTECs and HGECs were also cultured on 12-well Transwells® equipped with a PET membrane (0.4 μm pore size) in the absence and presence of 100 μl gelatin–fibrin coating (estimated 1 mm thick).

### RNA-seq

RNA isolation was performed utilizing Qiagen's miRNeasy mini kit according to manufacturer's protocol with minor modifications. Briefly, isolation was performed by injection of Qiazol lysing reagent through each channel on chip and then collection of the lysate. After homogenization *via* vortex mixing, the lysate was spun for 15 min at 4 °C to remove any unwanted gelatin–fibrin. Subsequent steps in the procedure followed the manufacturer's protocol, including the on-column DNase digestion steps. The quantity and quality of RNA samples was assessed using the standard sensitivity RNA fragment analysis kit on fragment analyzer (Agilent Technologies). All samples had an RNA integrity number >8 and were deemed of sufficient quality and quality for RNA-seq analysis. Samples were diluted to a final quantity of 200 ng per well of total RNA using the standard RNA analyzer kit on a fragment analyzer (Agilent Technologies). The TrueSeq Stranded mRNA LT sample prep kit (Illumina) was used to construct poly (A) selected RNA and then used for reverse transcription, generation of double stranded cDNA and subsequent library preparation and indexing to facilitate multiplexing. All libraries were quantified with the fragment analyzer using the standard sensitivity NGS kit (Agilent Technologies), pooled in equimolar concentrations and quantified with a Qubit fluorometer (ThermoFisher Scientific) with the DNA standard sensitivity kit (ThermoFisher Scientific). The library pool was further diluted to 1.8 pM and sequenced at >10 M paired end reads/sample using the high output reagent kit to 150 cycles on an Illumina NextSeq500. Three biological replicates were sequenced per sample.

### Data sharing

Data used in this paper has been deposited in the Gene Expression Omnibus (GEO) database, https://www.ncbi.nlm.nih.gov/geo (accession no. GSE198822).

### Data analysis

All samples were processed and analysed using bcbio-nextgen pipeline v1.1.3 (https://bcbio-nextgen.readthedocs.io) where salmon^22^ was used for expression quantification. bcbioRNASeq R object^23^ was created for normalization and differential expression analysis (using DESeq2).^24^ Cell type specific markers were taken from Young *et al.*^7^ where top 50 most significant marker genes from each cluster were selected for GSVA analysis.^25^ Gene Set Enrichment analyses was performed between (200801–201110) utilizing Enrichr (https://maayanlab.cloud/Enrichr/). Comparison of the top one-hundred upregulated genes with human tubulointerstitial biopsies was performed utilizing NephroSeq v4 (https://www.nephroseq.org/). Comparison analyses and prediction of upstream regulators and gene heatmaps of these upstream regulators were generated utilizing ingenuity pathway analysis (Version 57662101). PCA analysis was performed with counts normalised using variance stabilising transformation (vst) in the DESeq2 package.

## Disclosures

Miguel Carracedo, Babak Alaei, Maryam Clausen, Ryan Hicks, Graham Belfield, Magnus Althage, Annette Bak, Pernille B. L. Hansen and Julie Williams are all employees of AstraZeneca.

## Author contributions

Sanlin Robinson performed the *in vitro* experiments and contributed to the manuscript revision; Graham Belfield and Maryam Clausen performed the RNA-seq work and contributed to the manuscript revision; Babak Alaei performed the bioinformatics analysis and helped analyse the data; Miguel Carracedo analysed the data and wrote the manuscript; Ryan Hicks, Magnus Althage and Annette Bak contributed to the manuscript writing and final version; Jennifer A. Lewis, Pernille B. L. Hansen and Julie M. Williams conceived and supervised the study and contributed to writing the manuscript.

## Funding

This research was funded by AstraZeneca PLC and NCATS Tissue Chips 2.0 (NIH UH3TR002155) (SR and JAL).

## Conflicts of interest

Miguel Carracedo, Babak Alaei, Maryam Clausen, Ryan Hicks, Graham Belfield, Magnus Althage, Annette Bak, Pernille B. L. Hansen and Julie Williams are all employees of AstraZeneca.

## Supplementary Material

LC-023-D2LC00723A-s001

LC-023-D2LC00723A-s002

LC-023-D2LC00723A-s003
