# Supplementary figures and images for: 3D vascularised proximal tubules-on-a-multiplexed chip model for enhanced cell phenotypes
Source: Lab Chip. 2023 Jun 21;23(14):3226–37. doi: 10.1039/d2lc00723a (PMC10337267; doi:10.1039/d2lc00723a)

### PTEC culture in tubule over time

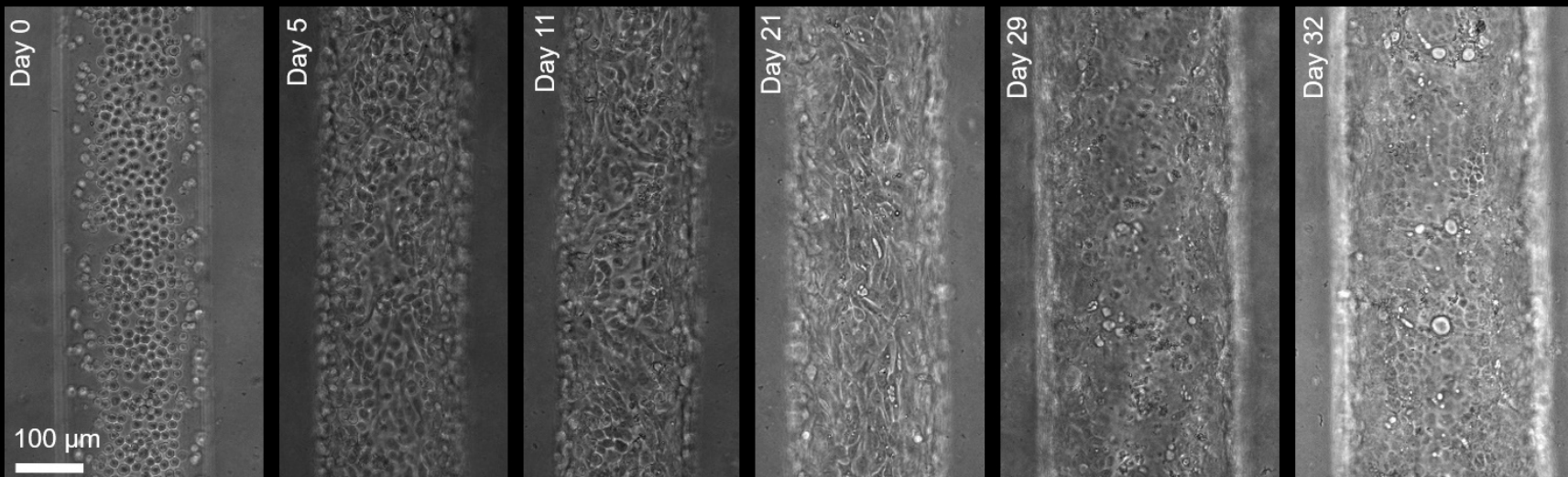

### HGECs added to second tubule on same chip on Day 29

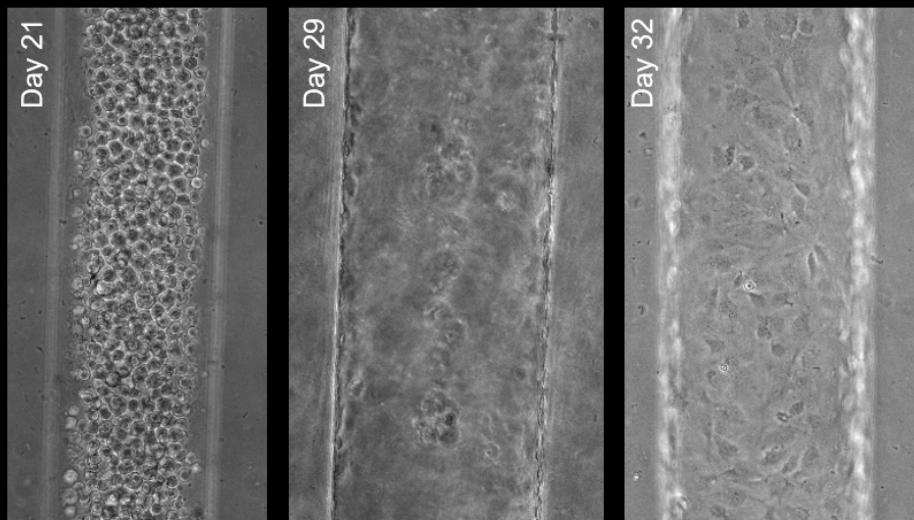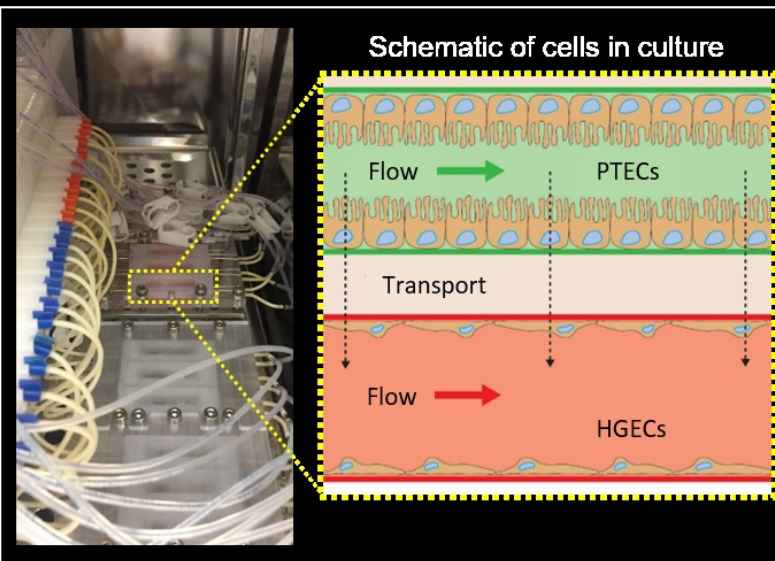

Supplement: LC-023-D2LC00723A-s002 [file LC-023-D2LC00723A-s002.pdf]

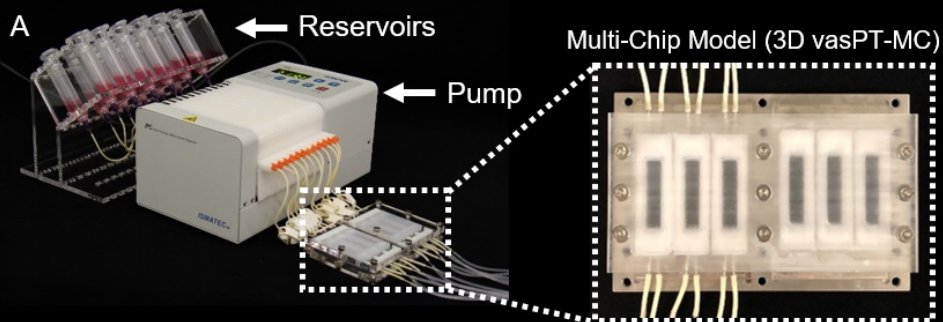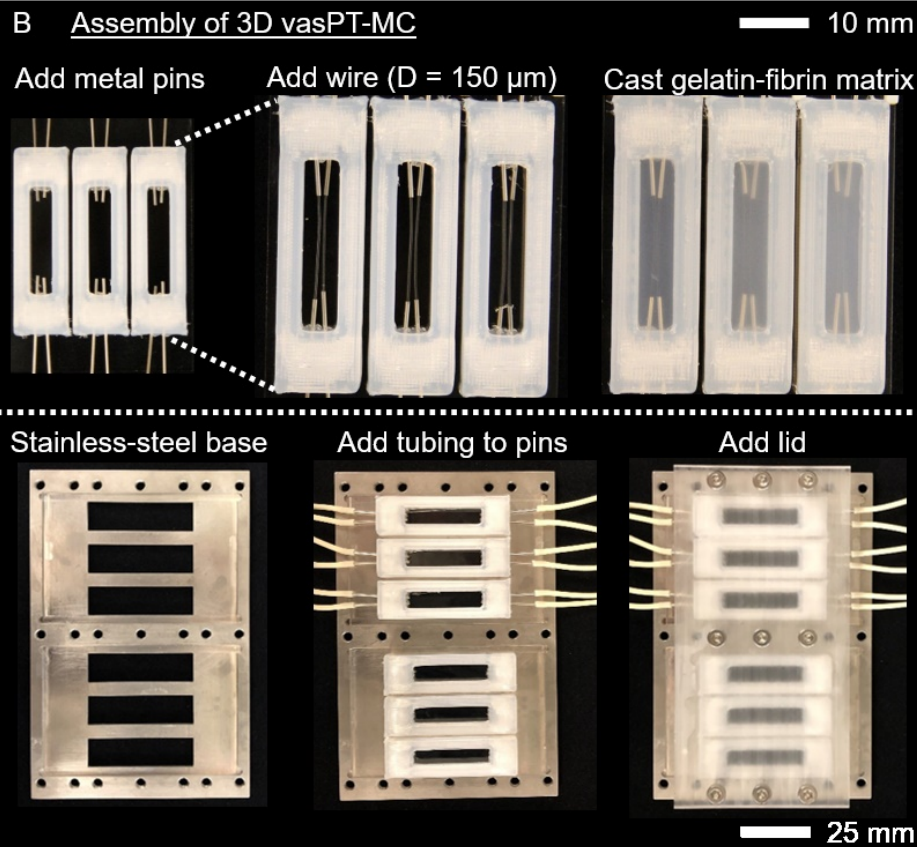

Supplement: LC-023-D2LC00723A-s003 [file LC-023-D2LC00723A-s003.pdf]
